# Genetic inactivation of the pancreatitis-inducible gene *Nupr1* impairs PanIN formation by modulating Kras^*G12D*^-induced senescence

**DOI:** 10.1038/cdd.2014.74

**Published:** 2014-06-06

**Authors:** D Grasso, M N Garcia, T Hamidi, C Cano, E Calvo, G Lomberk, R Urrutia, J L Iovanna

**Affiliations:** 1 Centre de Recherche en Cancérologie de Marseille (CRCM), INSERM U1068, CNRS UMR 7258, Aix-Marseille Université and Institut Paoli-Calmettes, Parc Scientifique et Technologique de Luminy, Marseille, France; 2 Molecular Endocrinology and Oncology Research Center, CHUL Research Center, Quebec City, QC, Canada; 3 Laboratory of Epigenetics and Chromatin Dynamics, Gastroenterology Research Unit, Departments of Biochemistry and Molecular Biology, Biophysics, and Medicine, Mayo Clinic, Rochester, MN, USA

## Abstract

Nuclear protein 1 (Nupr1), a small chromatin protein, has a critical role in cancer development, progression and resistance to therapy. Previously, we had demonstrated that Nupr1 cooperates with Kras^*G12D*^ to induce pancreas intraepithelial neoplasias (PanIN) formation and pancreatic ductal adenocarcinoma development in mice. However, the molecular mechanisms by which Nupr1 influences Kras-mediated preneoplastic growth remain to be fully characterized. In the current study, we report evidence supporting a role for Nupr1 as a gene modifier of Kras^*G12D*^-induced senescence, which must be overcome to promote PanIN formation. We found that genetic inactivation of Nupr1 in mice impairs Kras-induced PanIN, leading to an increase in *β*-galactosidase-positive cells and an upregulation of surrogate marker genes for senescence. More importantly, both of these cellular and molecular changes are recapitulated by the results of mechanistic experiments using RNAi-based inactivation of Nupr1 in human pancreatic cancer cell models. In addition, the senescent phenotype, which results from Nupr1 inactivation, is accompanied by activation of the FoxO3a-Skp2-p27^*Kip1*^-pRb-E2F pathway *in vivo* and *in vitro*. Thus, combined, these results show, for the first time, that Nupr1 aids oncogenic Kras to bypass senescence in a manner that cooperatively promotes PanIN formation. Besides its mechanistic importance, this new knowledge bears medical relevance as it delineates early pathobiological events that may be targeted in the future as a means to interfere with the formation of preneoplastic lesions early during pancreatic carcinogenesis.

Pancreatic ductal adenocarcinoma (PDAC) is a highly aggressive cancer with <5% survival after 5 years and a median survival of <6 months after diagnosis.^1^ PDAC progresses from precursor lesions named pancreas intraepithelial neoplasias (PanINs). In this regard, it has been firmly established that oncogenic mutations in *KRAS* behave as one of the earliest stimuli for the formation of PanINs.^2, 3^ These data are strongly supported by animal models, such as the *Pdx1-Cre; LSL-Kras*^*G12D*^ transgenic mice, in which the pancreas-specific expression of oncogenic Kras promotes PanIN occurrence^4^ and, at a lower frequency, pancreatic cancer. Thus, the role of Kras as an initiating cancer mutation is one of the best-established pathobiological mechanisms required for the development of pancreatic cancer. Noteworthy, however, during the initiation stage, pancreatic cells not only trigger protumoral processes but also cellular events that aim at counteracting transformation. One of these tumor-suppressive processes elicited by Kras activation is cellular senescence (oncogene-induced senescence). In the pancreas, the induction of senescence underlies the resistance of exocrine cells to oncogenic Kras-mediated transformation^5^ so as to prevent tumor promotion, which is often supported by common diseases such as chronic pancreatitis.^6^ Indeed, tissue injury, as it occurs in pancreatitis, weakens the defense mechanism posed by senescence leading to its bypass by exocrine cells, which can then readily form PanINs.^5^ Therefore, the molecular mechanism that supports the development of oncogene-induced senescence (OIS) needs to be fully elucidated, if we want to advance our understanding of pancreatic cancer development.

The nuclear protein 1 (Nupr1) is a basic helix–loop–helix molecule that is strongly induced by acute pancreatitis and several other cell stresses.^7, 8^ Nupr1 is also overexpressed in several types of human cancers, including PDAC. In this regard, the expression of genes that are targets for regulation by Nupr1 has been implicated in key protumorigenic pathways, including cell cycle regulation, matrix remodeling, autophagy, cell cannibalism and apoptosis inhibition.^9, 10, 11, 12, 13, 14, 15, 16^ Moreover, the fundamental role that Nupr1 has in pancreatic tumorigenesis is underscorded by recent results, which showed that, in mice, the oncogenic form of Kras^*G12D*^ is unable to promote PanINs in the absence of this chromatin protein,^17^ although the mechanisms responsible for this effect remain an area of active investigation. Consequently, we designed the current study with the aim of testing the hypothesis that Nupr1 cooperates with oncogenic Kras to induce PanIN formation by modulating the expression of gene networks that are necessary for bypassing senescence. To address this question, we characterized the effects that Nupr1 inactivation has on Kras-induced senescence using genome-wide expression profiling, as well as both cellular and molecular assays for this process. As a result of these experiments, we found that, indeed, the genetic inactivation of Nupr1 induces cellular senescence in exocrine pancreatic cells and reduces Kras-induced PanIN formation. At the molecular level, we demonstrated that this phenomenon is characterized by the upregulation of gene networks, which are known mediators of this phenomenon, by regulating at the G1/S transition. Taken together, these results provide mechanistic insights into how Nupr1 cooperates with Kras to promote the development of pancreatic preneoplastic lesions by discovering and characterizing a role for this pancreatitis-inducible protein in modulating cellular senescence. Thus, the new information emerging from this study has both mechanistic and biomedical implications for a better understanding of the pathobiology of pancreatic cancer.

## Results

### Inactivation of *Nupr1* by homologous recombination facilitates the development of Kras^*G12D*^-induced senescence *in vivo*

**Figure 1:**
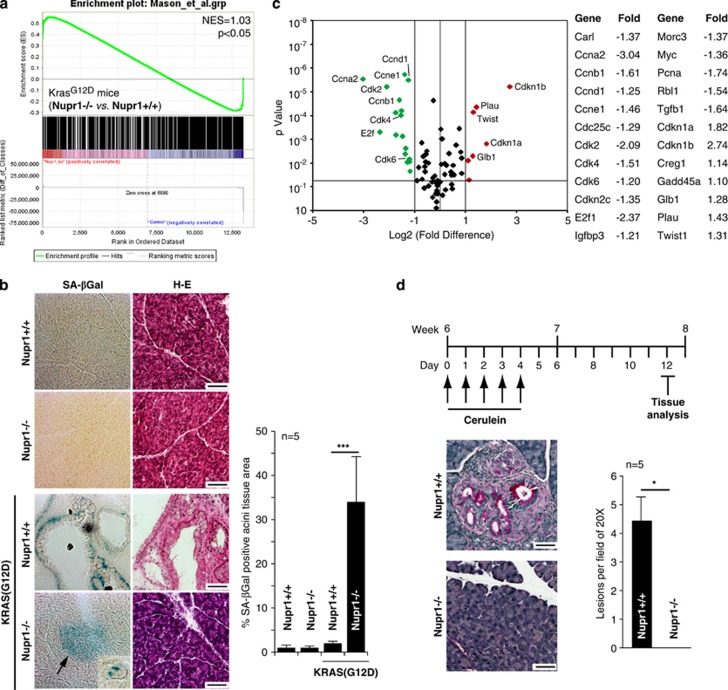
Nupr1 depletion triggers OIS in Kras^*G12D*^-expressing mice and prevents PanINs developments even after a pancreatitis injury. (**a**) GSEA analysis of DNA microarray from Nupr1^−/−^ *versus* Nupr1^+/+^ Kras^*G12*^-expressing pancreas. The figure shows Nupr1^−/−^ gene set enrichment in OIS- related genes (see text). (**b**) Serial frozen pancreas tissue sections with hematoxylin and eosin (H–E) and SA-*β*Gal staining showing senescent areas in Nupr1^−/−^ Kras^*G12D*^-expressing mouse pancreas tissue (arrow). (Inset) Nupr1^−/−^ Kras^*G12D*^-expressing mouse pancreas tissue depicts an example of morphologically normal acini presenting positive staining for SA-*β*Gal. Only low-grade PanINs show SA-*β*Gal-positive staining in Nupr1^+/+^ Kras^*G12D*^pancreas. No staining is observed in wild-type Kras animals nor in duct cells. (**c**) Volcano plot illustrating macroarray data performed on mRNA from Kras^*G12D*^-expressing pancreas from Nupr1^+/+^ or Nupr1^−/−^ mice. Red dots indicate upregulated genes in Nupr1^−/−^ animals, whereas green dots represent downmodulated genes. (**d**) Scheme of cerulein-induced pancreatitis (upper panel). H–E tissue analysis of pancreas after pancreatitis shows early induction of PanINs in Nupr1^+/+^ Kras^*G12D*^ mice and no lesions in Nupr1^−/−^ Kras^*G12D*^ animals (lower panel). Scale bar, 80 *μ*m

We have previously shown that deletion of the *Nupr1* gene in mice prevents Kras-induced PanIN development.^17^ However, the defined mechanisms underlying this phenomenon remain poorly understood. As Kras-driven neoplastic growth induction can result from the effects that this oncogene has on cell proliferation, apoptosis or senescence, we reasoned that the effects of Nupr1 on inhibiting PanIN formation may reflect at least one of these functions. Consequently, we began our studies by carefully examining expression profiles in RNA extracted from both Nupr1^+/+^ and Nupr1^−/−^, Kras^*G12D*^-expressing pancreata. We compared microarray data of pancreata derived from 5-week-old animals (*n*=3), as this developmental stage precedes the appearance of PanINs. Gene set enrichment analysis (GSEA) of these data sets indicated that deletion of Nupr1 modifies Kras^*G12D*^-associated transcriptional responses, in particular those linked to the regulation of two highly interdependent cellular phenomena, namely cell growth (Supplementary Table 1) and senescence (Figure 1a). This finding suggests that Nupr1 deletion impairs PanIN formation, at least in part, through an effect on the OIS. Consequently, we investigated the senescence status of both Nupr1^+/+^ and Nupr1^−/−^ Kras^*G12D*^-expressing pancreata by performing histochemistry for senescence-associated *β*-galactosidase activity (SA-*β*Gal) followed by morphometric analysis. Figure 1b shows that Nupr1 deletion associates with an increase in the areas of exocrine pancreatic tissue (mean 34±11%, *n*=5). Notably, SA-*β*Gal activity in Nupr1^−/−^ pancreata was detected as well-defined isolated patches of morphologically normal acinar cells. On the other hand, Nupr1^+/+^ Kras^*G12D*^-expressing pancreata display positive SA-*β*Gal staining in PanINs lesions, although not in morphologically normal acini (Figure 1b). As control, no SA-*β*Gal activity was detected in the pancreas from Nupr1^+/+^ and Nupr1^−/−^ mice that do not carry an activated form of Kras (Figure 1b), demonstrating that senescence correlates with the oncogenic activity of this small GTPase. Pathway-specific polymerase chain reaction (PCR) arrays demonstrate that the enhanced SA-*β*Gal staining in Nupr1^−/−^ pancreas is accompanied by the expression of well-characterized surrogate markers for senescence, including the downregulation of cell cycle progression-related genes and upregulation of p27^*Kip1*^ and p21 (Figure 1c). Thus, we decided to complement these studies by treating animals with cerulein, a cholecystokinin analog, which activates the Gq pathway, which synergizes with Kras so as to bypass senescence and induce cell growth during pancreatic carcinogenesis.^5^ Figure 1d shows that, in animals expressing wild-type Nupr1 alleles but not oncogenic Kras^*G12D*^, cerulein induced PanIN lesions as early as 8 weeks. In contrast, PanIN formation was impaired in the pancreata of Kras^*G12D*^-expressing Nupr1^−/−^ animals treated with cerulein (Figure 1d). Microscopic examination of the pancreas from these animals revealed a rather normal morphology and maintained high level of SA-*β*Gal-positive areas (mean 41±14%, *n*=4) (Figure 1d). Thus, Nupr1 deficiency appears to favor the establishment of Kras^*G12D*^-induced senescence in a manner that cannot be reversed by growth-synergizing stimuli, such as those induced by cerulein treatment.

### RNAi-mediated inactivation of Nupr1 recapitulates the development of Kras^*G12D*^-induced senescence in cultured human pancreatic cancer cells

**Figure 2:**
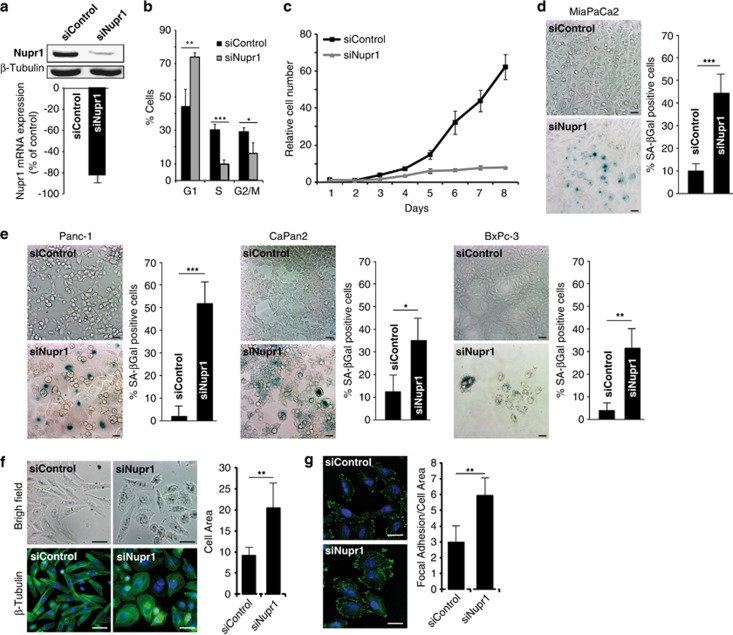
Nupr1 silencing triggers OIS in human pancreatic cancer cells. (**a**) Western blot and RT-qPCR of Nupr1 showing the effectiveness of specific siRNA in MiaPaCa2 cells. (**b**) Cytometric cell cycle analysis showing increase of G1-arrested cells by siNupr1 treatment in MiaPaCa2 cells. (**c**) MiaPaCa2 cell proliferation curve shows a significant stop in cell proliferation after siNupr1 silencing compared with siControl-treated cells. The relative cell number at each time point on the growth curves represents the mean±S.D. of triplicate normalized to the cell number at day 1. (**d**) SA-*β*Gal staining of MiaPaca2 cells. A very significant increase of senescent cells is observed after Nupr1 depletion. (**e**) SA-*β*Gal activity staining in Panc1, CaPan-2 and BxPC-3 cells treated with a siRNA against Nupr1. (**f**) Bright field and *β*-tubulin immunofluorescence of MiaPaCa2 cells treated with siNupr1 or siControl. Change in cell morphology and significant increase in cell size is observed in Nupr1-silenced cells. (**g**) Vimentin immunofluorescence showing significant increase of focal adhesions number in siNupr1-treated cells. Error bars±S.D.; *P<0.05, **P<0.01 and ***P<0.001. Scale bar, 10 *μ*m

To extend our *in vivo* results, we next explored the role of Nupr1 as a modifier of the effects of Kras^*G12D*^ in cultured human pancreatic cancer cells. We observed that Nupr1 is overexpressed in several pancreatic tumor cells such as MiaPaCa2, Panc1, CaPan2 and BxPC-3 cells (Supplementary Figure 1). Figures 2b–d show that after 5 days of siNupr1 treatment, Nupr1 downregulation arrests cell cycle progression (Figure 2b), inhibits cell proliferation (Figure 2c) and induces senescence-associated SA-*β*Gal activity in MiaPaCa2 cells (mean 43±15 *versus* 11±10, *P*<0.001) (Figure 2d). The reliability of this observation was further enhanced by obtaining similar results in three additional PDAC-derived cell lines, namely Panc1, CaPan2 and BxPC3 (Figure 2e). In addition, careful microscopic examinations reveal that siNupr1-treated pancreatic cancer cells adopt morphological features, which, when evaluated morphometrically, are compatible with those previously reported for cells undergoing OIS.^18^ These features include an increase in cell area (mean 20.3±8.5 *versus* 9.1±1.8, *P*<0.01), with a flat and rounded shape (Figure 2f), and larger number of focal adhesions (mean 6.0±1.2 *versus* 3.0±1.0, *P*<0.01) (Figure 2g).

As described previously, we observed that Nupr1 silencing increases apoptosis, in addition to senescence. Therefore, we used flow cytometry analysis to simultaneously quantify apoptosis and the SA-*β*Gal activity assay in the same cell population. As expected, siNupr1-treated cells presented a significant increase in the number of apoptotic cells (Supplementary Figure 2). Interestingly, the inhibition of apoptosis in siNupr1-treated cells using the pancaspase inhibitor zVAD results in an increase of SA-*β*Gal-positive cells (Supplementary Figure 2).

Thus, these experimental manipulations in cultured human pancreatic cancer cells recapitulate the results obtained in genetically engineered mice and, together, demonstrate that normal levels of Nupr1 expression are necessary for both bypassing OIS and modulating neoplastic cell growth.

### Nupr1 deficiency triggers OIS in cultured human pancreatic cancer cells through the activation of molecular pathways that regulate cell cycle

**Figure 3:**
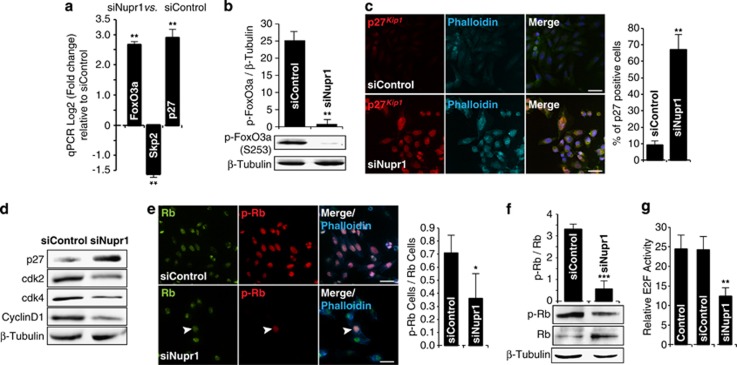
Nupr1 depletion triggers the FoxO3a-Skp2-p27^*Kip1*^ pathway. (**a**) By RT-qPCR, increase of FoxO3a, which negatively regulates Skp2, decrease of Skp2, which degrades p27^*Kip1*^, and consequently increases of p27^*Kip1*^ transcript is observed in Nupr1-depleted cells. (**b**) Phosphorylated FoxO3a, by western blot, in siNupr1 or siControl MiaPaCa2-treated cells. (**c**) Positive nuclear immunofluorescence of p27^*Kip1*^ is observed in siNupr1-treated cells. No signal is observed in cells upon siControl treatment. (**d**) Western blot shows the increase of p27^*Kip1*^ protein and decrease of Cdk2, Cdk4 and cyclin D1 in cells depleted of Nupr1. (**e**) Immunofluorescence of Rb (green) and phospho-Rb (Red) proteins in MiaPaCa2 cells. In siControl-treated cells, all cells present the phosphorylated form of Rb. On the contrary, only one cell (arrowhead) is positive for phospho-Rb in Nupr1-depleted cells. (**f**) Western blot showing the increase of the active hypophosphorylated form of Rb in MiaPaCa2 cells. (**g**) Reporter gene assay for E2F transcription factors. Significant decreased activity is observed when cells are silenced for Nupr1. Error bars±S.D.; *P<0.05, **P<0.01 and ***P<0.001. Scale bar, 10 *μ*m

As our data thus far suggested that Nupr1 inactivation allows OIS, we subsequently focused on characterizing molecular mechanisms underlying that effect. In this regard, the expression profile data described above reveals that Nupr1 inactivation increases *FoxO3a* expression,^19^ a transcription factor involved in cell cycle arrest, which is a key step for establishing the senescence phenotype.^19, 20, 21^ Experiments using RT-qPCR confirmed that siNupr1-treated pancreatic cancer cells show an increase of 2.7-fold±0.1 in the levels of FoxO3a mRNA (Figure 3a). Concomitantly, the treatment with siNupr1 decreased the expression of Skp2 (1.2±0.2-fold) (Figure 3a) and increased the expression of p27^*Kip1*^ (2.9±0.4-fold) (Figure 3a), both molecules that are part of a single growth-regulatory pathway downstream of FoxO3a.^22^ Moreover, we observed that FoxO3a increase was accompanied by hypophosphorylation of this protein, which has been previously observed to correlate with its activation and nuclear translocation (Figure 3b). In fact, the active nuclear form of FoxO3a downregulates Skp2, which in turn prevents the degradation of p27^*Kip1*^, thereby increasing the levels of this protein to arrest the cell cycle. Congruently, we find that the specific silencing of Nupr1 in pancreatic cancer cells increases the levels of the cyclin-dependent kinase (Cdk) inhibitor p27^*Kip1*^(Figures 3a, c and d). Therefore, these data demonstrate for the first time that Nupr1, FoxO3a, Skp2 and p27^*Kip1*^ act in a network to influence the transcriptional regulation of each other, suggesting that their cell biological functions may also be linked. We next investigated whether silencing of Nupr1 expression, when it occurs concomitantly with increased levels of p27^*Kip1*^, contributes to the cell cycle arrest given by Nupr1 siRNA treatment as shown in Figures 2b and c. We found that the upregulation of p27^*Kip*^ in siNupr1-treated cells is accompanied by a decrease in the levels of Cdk2, Cdk4 and cyclin D1, as well as Rb phosphorylation, and E2F promoter reporter activity, which are among the best characterized surrogates for p27^*Kip1*^-mediated proliferation arrest during the induction of senescence (Figures 3d–g). Hence, when combined, these data suggest that downregulation of Nupr1 results in FoxO3a overexpression, cell cycle arrest and senescence, a status that is characterized by activation of the Skp2-p27^*Kip1*^-Rb-E2F pathway.

### Genetic inactivation of Nupr1 *in vivo* has an impact on the regulation of the FoxO3a-Skp2-p27^*Kip1*^ pathway and the regulation of OIS

**Figure 4:**
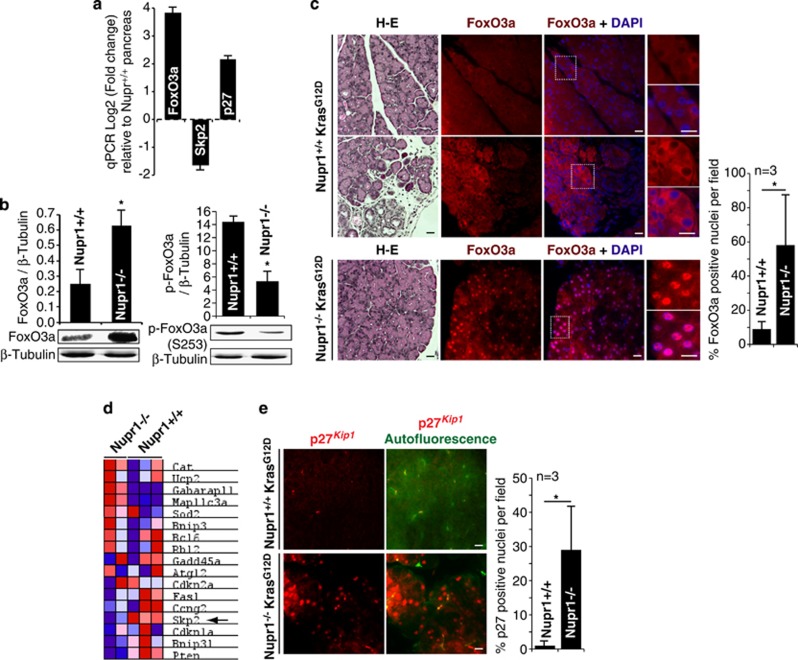
The FoxO3a-Skp2-p27^*Kip1*^ pathway is active in the Nupr1^−/−^ Kras^*G12D*^-expressing mouse pancreas. (**a**) RT-qPCR in Kras^*G12D*^-expressing mice pancreas. FoxO3a transcript is augmented in Nupr1^−/−^ compared with Nupr1^+/+^ pancreas. In the same way, a decrease in Skp2 and increase in p27^*Kip1*^ is detected in Nupr1-null animals compared with wild-type ones. (**b**) Western blots show an augmented and hypophosphorylated FoxO3a protein level in Nupr1^−/−^ Kras^*G12D*^ pancreas with respect to Nupr1^+/+^ Kras^*G12D*^. (**c**) Hematoxylin and eosin (H–E) staining. Immunofluorescence of FoxO3a in Nupr1^+/+^ Kras^*G12D*^-expressing pancreas shows low and cytoplasmic fluorescence either in normal (upper panel) or PanINs (middle panel) areas. On the other hand, Nupr1^−/−^ Kras^*G12D*^-expressing pancreas present a high and active nuclear fluorescence of FoxO3a (lower panel). (**d**) Heatmap of some FoxO3a target genes showing activation of several of them in Nupr1^−/−^ *versus* Nupr1^+/+^ Kras^*G12D*^-expressing animals. Moreover, the repression of Skp2 is denoted by an arrow. (**e**) p27^*Kip1*^ immunofluorescence with no detectable signal in Nupr1^+/+^ Kras^*G12D*^ mice and positive nuclear fluorescence in Nupr1^−/−^ Kras^*G12D*^ pancreas tissue. Error bars±S.D.; *p<0.05. Scale bar, 10 *μ*m

Owing to the potential mechanistic importance of the FoxO3a-Skp2-p27^*Kip1*^ pathway for Nupr1-mediated modulation of OIS revealed by our *in vitro* experiments, we subsequently investigated the status of this pathway *in vivo*. Both RT-qPCR and western blot analyses revealed an increase in the hypophosphorylated form of FoxO3a within the pancreas from Kras^*G12D*^-expressing Nupr1^−/−^ animals (Figures 4a and b). Immunofluorescence experiments reveal the presence of FoxO3a as a weak signal present in the cytoplasm of exocrine pancreatic cells from Kras^*G12D*^-expressing Nupr1^+/+^ animals (Figure 4c). In contrast, the pancreas from Kras^*G12D*^-expressing Nupr1^−/−^ mice show a strong nuclear signal for this protein (Figure 4c), which marks the active form of this transcription factor. Interestingly, the active FoxO3a signal was distributed in a patched pattern similar to the one given by SA-*β*Gal staining (Figure 1b). Consistent with this notion, our expression profiles, described in Figure 4d, reveal that pancreas tissue from Kras^*G12D*^-expressing Nupr1^−/−^ mice show significant changes in the expression of Foxo3a target genes, which also include the downregulation of Skp2 (Figure 4d). In addition, although immunofluorescence for p27^*Kip1*^ appears negative in the pancreas of Kras^*G12D*^-expressing Nupr1^+/+^ animals, the staining for this protein is readily positive in the glandular tissue of Kras^*G12D*^-expressing Nupr1^−/−^ mice adopting a patched pattern throughout the tissue (Figure 4e). In aggregate, these results demonstrate that the FoxO3a-Skp2-p27^*Kip1*^ pathway, first defined by our *in vitro* mechanistic experiments in cultured pancreatic cancer cells (Figure 3), associates with the Nupr1^−/−^ genotype *in vivo* giving rise to OIS with a concomitant impairment in PanIN development.

## Discussion

Elegant studies, primarily performed during the past three decades, have focused on defining how pancreatic cancer progresses through the successive transformation of normal exocrine cells into those that have the ability to form PanINs with various degrees of malignant potential. Additionally, the collective works of many laboratories have clarified that, at a molecular level, the progression of these lesions is caused by the accumulation of genetic mutations in a subset of well-known oncogenes and tumor suppressors. Although the knowledge derived from these investigations have been remarkably helpful in providing a better mechanistic understanding of pancreatic cancer development and provide potential markers for diagnosis as well as promising therapeutic targets, it has mostly remained genetic centric. Actually, oncogenic activation by mutation promotes cell growth and transformation, although in some cases these oncogenes result in insufficient senescence because the simultaneous antitransformation mechanisms are activated by the cells to counteract the oncogenic effect; among these mechanisms the major one is the OIS. Consequently, mechanisms regulating OIS may influence transformation. In pancreatic cancer, one of the mechanisms regulating senescence is pancreatitis, one of the better identified factors promoting transformation by oncogenic Kras.^5^ Unfortunately, the genes and mechanisms, which regulate OIS and thereby promote transformation by pancreatitis, remain unidentified. Thus, this work is very relevant to the field as we demonstrated that the pancreatitis-induced protein Nupr1 acts in concert with the mutated Kras to facilitate the progression of pancreatic cancer, at least in part, through permissive effects for bypassing senescence.

Serrano *et al.*^18^ first reported the potential of oncogenic Ras to induce a permanent cell growth arrest in non-pancreatic cell populations. Today we know that oncogenic Ras promotes cell cycle arrest, which accompanies morphological and molecular changes in affected cells characteristic of replicative senescence. In the pancreas, the expression of Kras^G12V^ in exocrine cells initially induces cell proliferation giving rise to PanIN precursor lesions, although at a later point, these cells subsequently stop dividing. At this point, the precursor lesions display both cellular and molecular changes, which are characteristic of senescence and their progression to cancer, an outcome that requires bypassing of this cellular mechanism. Bypassing senescence has been proposed to result from additional molecular defects that accompany Kras mutations.^23^ This knowledge has fueled an active area of investigation from which emerging data suggest that, through the regulation of cell cycle-related genes, transcription factors and chromatin proteins have a pivotal role in the regulation of this process. Notably, previous investigations both in mouse and human have demonstrated that Nupr1 cooperates with Kras not only during PanIN formation and PDAC development but, in addition, these two proteins arm frank pancreatic cancer with a more aggressive phenotype, characterized with poor survival and resistance to chemotherapy.^24^ Thus, the current study not only identifies for the first time senescence as a cellular mechanism linking the functions of these two proteins but also adds Nupr1 to the growing group of stress-associated proteins involved in the regulation of this process.

Interestingly, we also found that Nupr1 depletion induces OIS in several human pancreatic cancer cells lines including the BxPC-3 (Figure 2e), which express a wild-type version of Kras protein.^25^ These data suggest that the OIS due to Nupr1 silencing is not exclusive of oncogenic Kras mutation, but rather a more intrinsic cellular mechanism against cell transformation. This concept is consistent with the flow cytometry data (Supplementary Figure 2) where apoptosis inhibition in Nupr1 silenced increases the percentage of senescent cells. This finding suggests that senescence is an alternative to apoptosis in response to transformation or uncontrolled proliferation and differences between the apparently two populations is just the alternative path chosen by each cell. Thus, we are optimistic that future investigations, using complementary cellular and biochemical approaches in cells, mice and human, such as the ones shown in the current study, will shed light on additional mechanisms that dictate why some cells are prone to apoptosis and others to senescence.

Owing to the fact that OIS acts as a crossroad in the path toward cancer development, we sought to illuminate the molecular underpinning of this cellular mechanism. In this task, we were guided by the hypothesis that, because of its function as chromatin protein, Nupr1 may modify the function of Kras by regulating the expression of senescence-associated gene networks. We initially tested this hypothesis, using state-of-the-art methodology, by performing a genome-wide expression profiling. Fortunately, the results of these experiments provided the first available evidence supporting the role of Nupr1 in the regulation of genes involved in cell senescence and growth, for instance, FoxO3a. Mechanistically, we found that Nupr1 downregulates *FoxO3a* expression at least in part by maintaining its promoter hypermethylated through a pathway involving Dnmt1 (manuscript in preparation). It has been demonstrated that FoxO3a in re-expressed in neuroblastoma cells treated with the DNA methylation inhibitor 5-aza-2-deoxycytidine.^26^ Moreover, the same inhibitor was related to dephosphorylation and nucleus translocation in acute leukemia.^27^ This suggests the DNA methylation regulation of *FoxO3a* gene in cancer. In our unpublished data, we found that Nupr1 silencing induces a strong reduction in Dnmt1 expression which in turn allows a hypomethylated state of FoxO3a promoter and by consequence to its expression. Foxo3a is found to be a transcriptional repressor of *Skp2* gene expression by directly binding to the *Skp2* promoter, thereby inhibiting Skp2 protein expression, which in turn promotes p27^*Kip1*^ stability.^22^ Also, p27^*Kip1*^ is a key regulator of cell cycle arrest and senescence.^28^ These observations were function validated using a robust battery of cellular and biochemical assays, which led us to uncover the FoxO3a-Skp2-p27^*Kip1*^ axis as an example of a cell cycle arrest effector pathway. However, in spite of the well-known role of this cell cycle inhibitor cascade in the regulation of senescence, we cannot exclude that other molecules, whose expression is modified by Nupr1, may contribute to the establishment and/or maintenance of this phenotype. Indeed, functional alterations in Nupr1 have been associated with several malignancies including breast, cholangiocarcinoma, colon cancer, prostate, bladder and lung. Thus, it remains possible that the observations resulting from the current study have a wider application than previously anticipated. These considerations raise optimism that this knowledge will fuel future studies aimed at shedding light onto the function of Nupr1 as a regulator of OIS in other organs and consequently have an impact on cancer development.

In conclusion, when combined, the results of the experiments reported in this article support the notion that gene expression changes mediated by the pancreatitis-induced protein Nupr1 modulate the function of well-characterized pancreatic oncogenes, in which mutational activation occurs early during the development of pancreatic cancer. In light of the reduced success of previous attempts to correct genetic alterations in pancreatic cancer through gene therapy, these considerations also highlight the possibility that early intervention against stress-induced proteins may be more beneficial for the management of this cancer and likely other malignancies, which are known to associate with Kras mutations.

## Materials and Methods

### Animals

Nupr1^−/−^ mice bear a homozygous deletion of exon 2 of the *Nupr1* gene and were reported previously.^29^ These mice are viable and fertile and exhibit normal pancreatic development, although they are more sensitive to systemic lipopolysaccharide and experimental pancreatitis.^30^ The *Pdx1-cre;LSL-Kras*^*G12D*^ mice were provided by R Depinho (Dana-Faber Cancer Institute, Boston, MA, USA) and resulted from crossbreeding of the following strains: *Pdx1-Cre*^31^ and *LSL-Kras*^*G12D*^.^32^ Pancreatitis was induced by intraperitoneal administration of cerulein (Sigma-Aldrich, St. Louis, MO, USA) at 250 *μ*g/kg of body weight for five consecutive days, followed by 1 week of recovery. Because animals are from different genetic backgrounds, we systematically used littermate control and experimental mice. Mice were kept within the Experimental Animal House of the *Centre de Cancérologie de Marseille* (CRCM) pole Luminy, following institutional guidelines.

### DNA microarray

Total RNA was isolated and reverse transcribed for hybridization to the Mouse Gene 1.0 ST Array (GeneChip; Affymetrix, Santa Clara, CA, USA) as described previously.^33^ Arrays were processed using the Affymetrix GeneChip Fluidic Station 450 (protocol EukGE-WS2v5_450) and scanned using a GeneChip Scanner 3000 G7 (Affymetrix). GeneChip Operating Software (Affymetrix GCOS v.1.4) was used to obtain chip images, with quality control performed using the AffyQCReport software (Bioconductor, Berkeley, CA, USA). GSEA was performed on the Board Institute Platform^34, 35^ and statistical significance (false discovery rate) was set at 0.25. Microarray data are available from Gene Expression Omnibus (National Center for Biotechnology Information, Bethesda, MD, USA) under the accession number GSE45232.

### Cell culture

MiaPaCa2, Panc1, CaPan2 and BxPC-3 cells were obtained from ATCC (Manassas, VA, USA) and maintained in DMEM (Invitrogen, Carlsbad, CA, USA) supplemented with 10% FBS at 37 °C with 5% CO_2_. INTERFERin reagent (Polyplus-transfection, New York, NY, USA) was used to perform siRNA transfections according to the manufacturer's protocol. Scrambled siRNA targeting no known gene sequence was used as negative control. The sequences of Nupr1-specific siRNA (siNupr1 r(GGAGGACCCAGGACAGGAU)dTdT and siNupr1 no. 2 r(AGGUCGCACCAAGAGAGAA)dTdT) were previously reported.^36^

### Histology and immunofluorescence

Pancreatic sections were fixed in 4% paraformaldehyde and paraffin embedded. H&E staining and immunofluorescence were performed using standard procedures. Sections were probed with primary antibodies: anti-p27 and anti-Rb polyclonal antibodies were from Santa Cruz Biotechnologies (Santa Cruz, CA, USA); the FoxO3a and phospho-Rb (Ser807/811) monoclonal antibodies were from Cell Signaling (Danvers, MA, USA); and the anti-*β*-tubulin polyclonal and anti-vimentin monoclonal antibodies were from Sigma-Aldrich. Alexa Flour 488 and 594 (Invitrogen) were used as secondary antibodies. Samples were mounted in ProLong Antifade Reagent with DAPI (Invitrogen) and examined in an Eclipse 90i Nikon microscope (Nikon Instruments Europe B.V., Champigny-sur-Marne, France).

### SA-*β*-galactosidase activity

Pancreas cryosections from Nupr1^−/−^ and Nupr^+/+^ Kras^*G12D*^ mice or cells cultured on glass coverslips were tested for SA-*β*Gal activity using the Senescence *β*-galactosidase Staining Kit (Cell Signaling) according to the manufacturer's protocol.

### RT-qPCR

Pancreatic RNAs from Nupr1^−/−^ and Nupr^+/+^ Kras^*G12D*^ mice were prepared immediately after dissection following Chirwin's protocol.^37^ RNA from cells was prepared using Trizol reagent (Invitrogen) and reverse transcribed using Go Script (Promega, Madison, WI, USA) according to the manufacturer's instructions. Real-time quantitative PCR was performed in a Stratagene Cycler (La Jolla, CA, USA) using Takara reagents (Shiga, Japan). Primers sequences are available upon request. For Figure 1c, Mouse Cellular Senescence RT2 Profiler PCR array (Qiagen, SA Biosciences, Frederick, MD, USA) was used according to the manufacturer's instructions.

### Immunoblotting

Protein extraction was performed on ice using total protein extraction buffer: 50 mM HEPES (pH 7.5), 150 mM NaCl, 20% SDS, 1 mM EDTA, 1 mM EGTA, 10% glycerol, 1% Triton, 25 mM NaF, 10 *μ*M ZnCl_2_ and 50 mM DTT. Before lysis, protease inhibitor cocktail at 1:200 (Sigma-Aldrich; NUPR1340), 500 *μ*M PMSF, 1 mM sodium orthovanadate and 1 mM *β*-glycerophosphate were added. Protein concentration was measured using a BCA Protein Assay Kit (Pierce Biotechnology). Protein samples (80 *μ*g) were denatured at 95 °C and subsequently separated by SDS-PAGE gel electrophoresis. After being transferred to nitrocellulose, the membrane was blocked with 1% BSA, and then the samples were probed with primary antibody, followed by a horseradish peroxidase-coupled secondary antibody. Primary antibodies used were as follows: anti-p27, anti-Cdk2, anti-Cdk4, anti-cyclin D1 and anti-Rb polyclonal antibodies were from Santa Cruz Biotechnologies; anti-Nupr1 monoclonal antibody was homemade and described previously;^17^ FoxO3a monoclonal, phospho-FoxO3a (Ser253) polyclonal and phospho-Rb (Ser807/811) monoclonal antibodies were from Cell Signaling; anti-*β*-tubulin polyclonal and anti-vimentin monoclonal antibodies were from Sigma-Aldrich. Image acquisition was made in Fusion FX image acquisition system (Vilber Lourmat, VWR, Fontenay-sous-Bois, France) and bands were quantified using the ImageJ software (NIH, Bethesda, MD, USA).

### Reporter gene assay

The reporter assays were performed with Luciferase Assay System (Promega) according to the manufacturer's instructions. Cells were plated in 24-well plates and the following day co-transfected with 200 ng of pGl2-3 × E2F and 20 ng of pRL-SV40 plasmids. The luciferase reporter activity of each sample was normalized against the internal control activity of *Renilla* luciferase developed with coelenterazine (Sigma-Aldrich). Each sample was determined in triplicate. The results represent means±S.E. from three experiment runs.

### Flow cytometry

Cell cycle analysis was performed by standard propidium iodide staining protocol on a FACSCalibur flow cytometer (BD Biosciences, San Diego, CA, USA). SA-*β*Gal activity was performed using the Quantitative Cellular Senescence Assay Kit (Cell Biolabs, San Diego, CA, USA). Apoptosis was measured with the Membrane Permeability/Dead Cell Apoptosis Kit with YO-PRO-1 and PI Kit (Molecular Probes-Life Technologies, Eugene, CA, USA). Acquisition of 50 000 events per sample was made in a MACSQuant-VYB (Miltenyi Biotec, Surrey, UK). Data analysis was performed using the FlowJo (Treestar, Ashland, OR, USA) software.

### Statistics

Statistical analyses were performed using the unpaired two-tailed Student's *t*-test, and non-normal distribution, unpaired data were obtained using the Mann–Whitney test. All tests of significance were two-tailed and the level of significance was set at 0.05. Values are expressed as mean±S.E.M. RT-qPCR data are representative of at least three independent experiments with technical duplicates completed.

## Abbreviations

Nupr1: nuclear protein 1
PanIN: pancreas intraepithelial neoplasias
PDAC: pancreatic ductal adenocarcinoma
SA-*β*Gal: senescence-associated *β*-galactosidase
PCR: polymerase chain reaction
Cdk: cyclin-dependent kinase
GSEA: gene set enrichment analysis
