# Supplementary figures and images for: Genetic inactivation of the pancreatitis-inducible gene Nupr1 impairs PanIN formation by modulating KrasG12D-induced senescence
Source: Cell Death Differ. 2014 Jun 6;21(10):1633–41. doi: 10.1038/cdd.2014.74 (PMC4158688; doi:10.1038/cdd.2014.74)

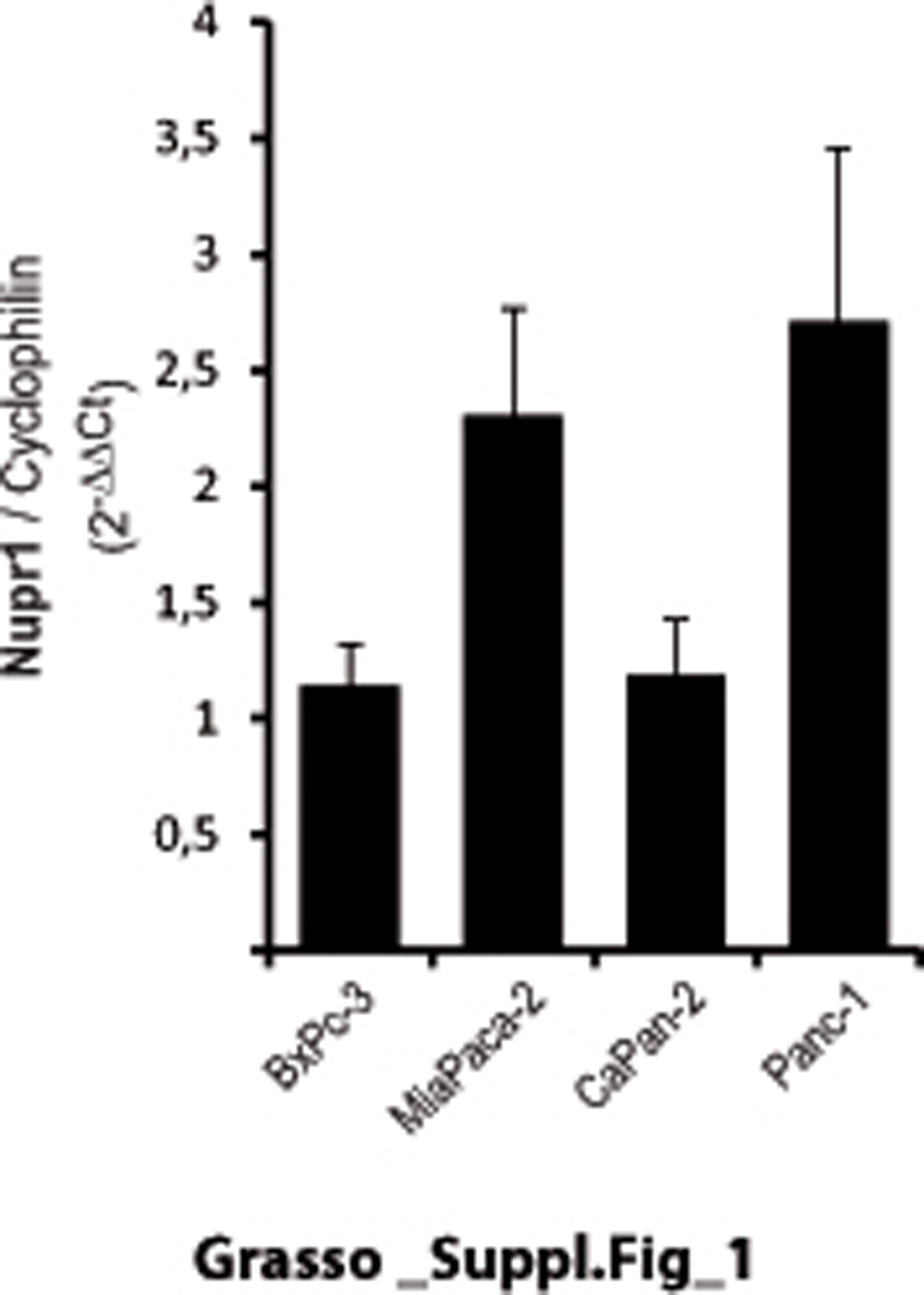

Supplement: Supplementary Figure 1 [file cdd201474x1.tif]

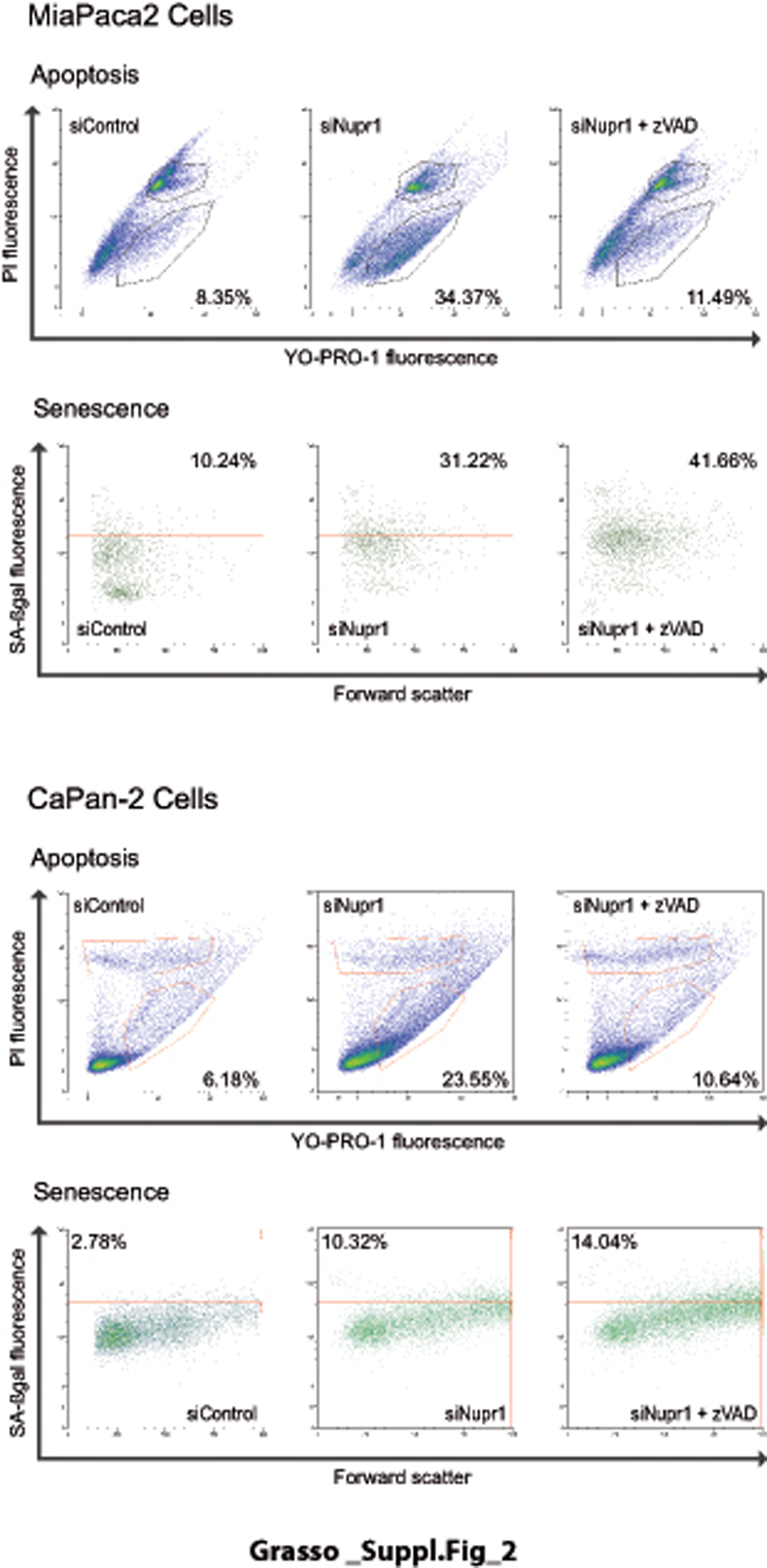

Supplement: Supplementary Figure 2 [file cdd201474x2.tif]
