# Supplementary material for: Genetic inactivation of the pancreatitis-inducible gene Nupr1 impairs PanIN formation by modulating KrasG12D-induced senescence
Source: Cell Death Differ. 2014 Jun 6;21(10):1633–41. doi: 10.1038/cdd.2014.74 (PMC4158688; doi:10.1038/cdd.2014.74)
Supplement: Supplementary Figure Legends [file cdd201474x3.doc]

**Supplementary Figure Legends**

**Supplementary Figure 1.**

Quantitative RT-qPCR showing Nupr1 transcript expression among different human pancreatic cancer cells lines. The housekeeping gene Cyclophilin was used as internal control in order to the normalizing of values.

**Supplementary Figure 2.**

Flow cytometry of MiaPaCa2 and CaPan2 cells upon siNupr1 or siControl treatments. For both cell lines an increase of apoptotic cells is observed under Nupr1 depletion. Additional apoptosis inhibition on siNupr1 treated cells (siNupr1 + zVAD) increases the senescent cells number.
